# Presence of the 54-chromosome common vole (Mammalia) on Olkhon Island (Lake Baikal, East Siberia, Russia), and the occurrence of an unusual X-chromosome variant

**DOI:** 10.3897/CompCytogen.v5i5.1720

**Published:** 2011-12-22

**Authors:** S.V. Pavlova, A.V. Tchabovsky

**Affiliations:** 1 A.N. Severtsov Institute of Ecology and Evolution, Russian Academy of Sciences, 33 Leninskiy pr., 119071 Moscow, Russia

**Keywords:** chromosome sibling species, common voles, *Microtus arvalis* group, *Microtus rossiaemeridionalis*, Lake Baikal, X-chromosome

## Abstract

We report a new finding of the 54-chromosome sibling species of the common vole in East Siberia - the first description from Olkhon Island (Lake Baikal). The karyotype of a male specimen revealed by routine staining and C-banding demonstrates the unambiguous presence of *Microtus rossiaemeridionalis* Ognev, 1924 (recently often regarded as as junior synonym of *Microtus levis* Miller, 1908). Comparison with conspecific specimens from the European part of the species range (from the left bank of the river Volga) shows that the vole of the island population has a smaller X-chromosome due to a reduced quantity of C-positive heterochromatin. This is just the third example of this type of X-chromosome variant with previous cases on an Arctic island (Svalbard) and the West Siberian lowland (Novosibirsk) and the only one on a lake island. Although *Microtus rossiaemeridionalis* is largely monomorphic in its karyotype, our data show that one specific type of X-chromosome variant is remarkably widespread, though rare.

## Introduction

The investigation of intraspecific variability of chromosomes is one of the traditional approaches to study evolutionary processes. Comparative karyological investigations of new and especially extreme localities of widely distributed species are of particular interest (Král et al. 1980).

Since the description of two sibling species in the common vole differing in diploid numbers (2n=46 in *Microtus arvalis* Pallas, 1779 and 2n=54 in *Microtus rossiaemeridionalis* Ognev, 1924) (Meyer et al. 1969, 1972, Malygin and Yatsenko 1986, Common Vole: The sibling species 1994) the vast range of the species previously known as *Microtus arvalis* has been revised to demarcate areas occupied by these new taxa (e.g. Shenbrot and Krasnov 2005). Among karyotypic data on *Microtus rossiaemeridionalis*, represented localities in its European part of the range (e.g. Král et al. 1980, Common Vole: The sibling species 1994, Meyer et al. 1996) outnumber those from Siberia, where data from no more than a dozen scattered sites are available from the huge Asian territory within the boundaries of the Russian Federation and Kazakhstan (Meyer et al. 1996, Yakimenko and Kryukov 1997).

Here we report one of the easternmost findings of the 54-chromosome karyotype for *Microtus rossiaemeridionalis*, from an isolated population on Olkhon Island in Lake Baikal.

## Materials and Methods

Small mammal trapping was conducted in July 2008 in Irkutsk province, East Siberia, on the western shore of Lake Baikal and on Olkhon Island that was separated by a narrow channel (2-5 km in width) from the mainland. A sole adult male of the common vole was live-trapped on Olkhon Island about 800 m from the village of Khalgai (53°42'14"N; 107°31'32"E) at the edge of larch – pine forest, bounded by steppe habitat.

**Figure 1.**
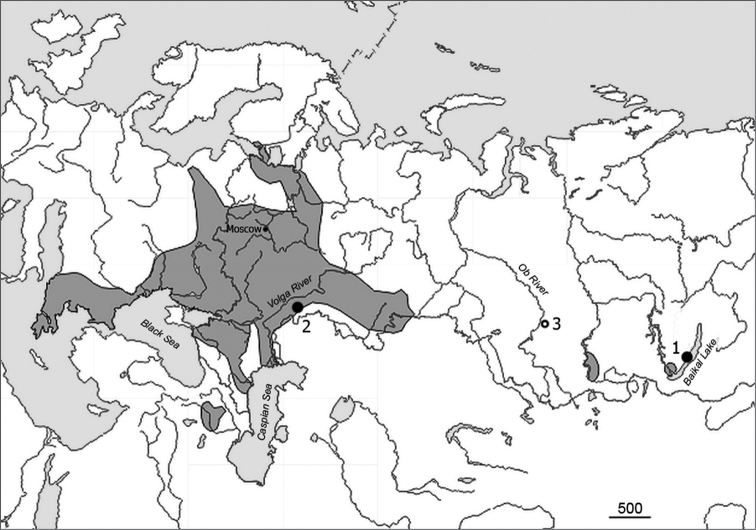
Map showing collection sites for the 54-chromosome (Sibling) vole under study. Black circles – our data (**1** Olkhon isl., Lake Baikal **2** Dyakovka, left bank of Volga River.) Open circle – most recent Siberian finding **3** Novosibirsk vic., right bank of Ob River. Colored are the territories where verified findings (karyotype, allozymes) were obtained from.

For the cytogenetic comparison, fresh chromosome preparations were prepared in a similar way for 3 specimens, 2 males and 1 female, collected in March 2011 in the European part of the range of the common vole (village of Dyakovka on the left bank of the river Volga, Saratov province: 50°42'54"N; 46°45'52"E). These animals were caught using live-traps on the bushy slope of the right bank of the river Yeruslan, about 400 m from the village and about 2.5 km from the Dyakovsky Forest (Fig. 1). In addition to our data, a recent Siberian collection site (Novosibirsk, West Siberia) is indicated in the distribution map of the *Microtus rossiaemeridionalis* taken from the official web-site of A.N. Severtsov Institute of Ecology and Evolution (http://www.sevin.ru/vertebrates/).

Materials for cytogenetic and further molecular analyses were fixed in the field following a standard protocol (Bulatova et al. 2009), while the skull and postcranial skeleton were deposited in the Laboratory of Historical Ecology of the A.N. Severtsov Institute.

Standard mitotic and meiotic chromosome preparations were obtained in the field from the bone marrow and from testes following Ford and Hamerton (1956) with some modifications (Bulatova et al. 2009) and Williams et al. (1971), respectively, and then analyzed in the laboratory under a light microscope. Routine Giemsa staining and the C-banding technique of Sumner (1972) were used to define the karyotype.

## Results

**Figure 2a-b.**
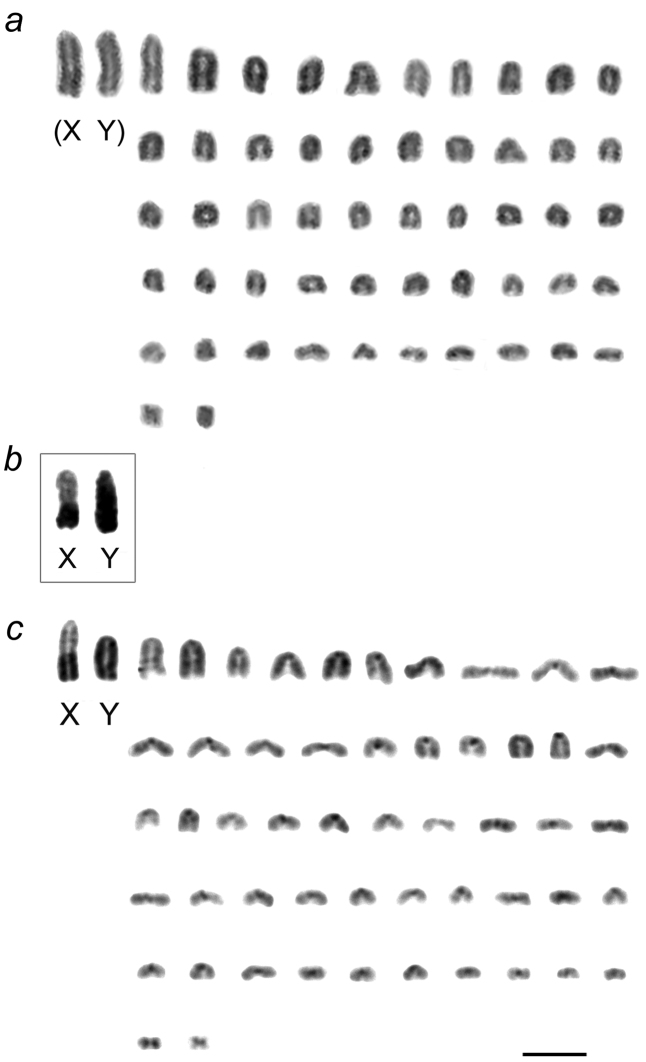
Mitotic chromosomes of East Siberian and East European *Microtus rossiaemeridionalis*: **a**conventionally stained chromosomes of the male from Olkhon Island arranged by size, with provisional identification of the XY sex chromosome pair, based on chromosome length **b** C-banded sex chromosomes of the same individual **c** C-banded chromosomes of a male from the east bank of the river Volga. Bar=10 µm.

The four voles examined had the same chromosome number 2n=54 and identical autosomal karyotype supplemented by the typical sex chromosomes complement – XX in females, XY in males. All chromosomes but the smallest pair of metacentric autosomes were acrocentric (NF=56). Routine staining was ineffectual in identifying more than two pairs of large acrocentrics and the small metacentric pair from the morphologically homogeneous group of medium to small acrocentrics. After C-banding, the centromeres of all autosomes were positively C-stained. The two largest elements with additional C-blocks of heterochromatin were classified as the pair of sex chromosomes, the Y being totally heterochromatic and the X carrying a large telomeric block (Fig. 2). The X chromosome was always the largest element in the complement and the Y the next largest, but close in size to the largest autosome.

**Figure 3.**
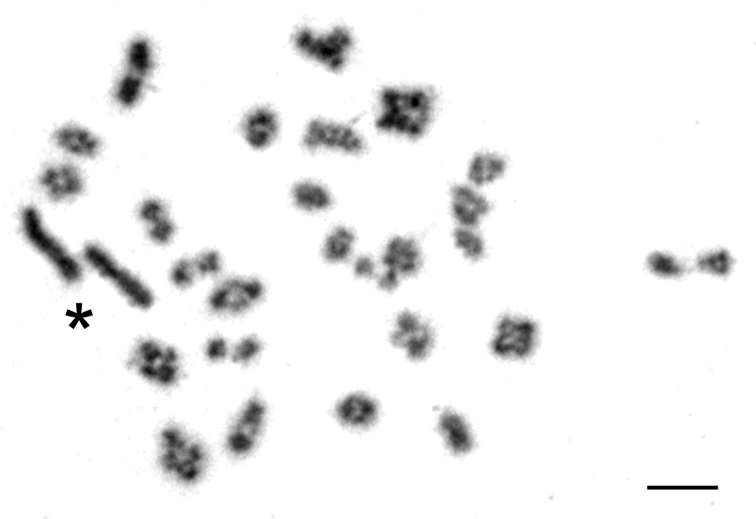
Meiotic spread with 26 autosomal bivalents and the characteristic asynaptic configuration of the sex chromosomes (star) from a male common vole of Olkhon Island (2*n*=54). Bar=10 µm.

However, the length of the X chromosome varied between voles from the two geographically distant regions. In the male from Olkhon Island both sex chromosomes looked alike in routinely stained karyograms and did not exceed considerably in size the largest autosome (Fig. 2a). They differed each from the other only by C-banding, and in this case the distal heterochromatic block marking the X occupied less than a half of its total length (Fig. 2b). Similar proportions in length of the sex chromosomes were seen in meiotic plates of this individual showing X and Y stick configuration. Autosomes formed bivalents during meiosis whereas sex chromosomes remain asynaptic (Fig. 3).

In stark contrast, in voles from the European sample the X was larger than either the Y or the larger autosomes, and this can only be due to a larger amount of telomeric heterochromatin occupying the distal half of the X chromosomes in males (Fig. 2c) as well as in a female studied.

## Discussion

The chromosomal characteristics of the specimens studied are consistent with the karyotypic features of the 54-chromosome sibling species of the common vole, a member of the *Microtus arvalis* group (Common Vole: The sibling species 1994). Two chromosomal sibling species of common voles, *Microtus arvalis* s. str. (2n=46) and *Microtus rossiaemeridionalis* (2n=54), can be recognized simply by the diploid number, wherever they occur. Localities of their separate or common distribution have been progressively sampled since the first description of the karyotypes in European Russia (Meyer et al. 1969) and have been many times updated, with the most comprehensive lists having been provided by Král et al. (1980), Common Vole: The sibling species (1994) and Meyer et al. (1996). For the 54-chromosome species, the first descriptions were obtained from the East European Plain and Caucasus, but there were few from neighboring Asian regions. In particular, Meyer et al. (1996) listed two geographic sites in Kazakhstan and two more in southern and eastern Siberia which indicated the border of species distribution eastwards. The most eastern finding was from Mount Khashkai, about 250 km to the west of Lake Baikal, Nukuty Distr., Irkutsk Prov. (ca. 53°40'N; 102°30'E, inferred from the map). Unfortunately, no details of those karyotypes were provided. Also there are no karyotypic details of the few individuals of *Microtus rossiaemeridionalis* previously obtained from Saratov Province (including the village of Dyakovka) for the electrophoretic analysis of haemoglobin in the blood (Tikhonova et al. 2005).

It is interesting that although the ranges of the two sibling species, *Microtus arvalis* and *Microtus rossiaemeridionalis*, significantly overlap in Eastern Europe and in their Asian parts; these species have been found, in general, to be separated in Siberia and Kazakhstan (Meyer et al. 1996). Since the 1990s, however, the 54-chromosome karyotype was detected in common voles from Novosibirsk (Yakimenko and Kryukov 1997, Mazurok et al. 1995, Fig.1), even though only 46-chromosome *Microtus arvalis* specimens were reported in earlier studies from the same geographical region (Král and Liapunova 1975, Král et al. 1980)

Further chromosomal studies added only a few occurrence sites for *Microtus rossiaemeridionalis* in Kazakhstan (Kovalskaya 1994), Western Siberia (close to Novosibirsk; Yakimenko and Kryukov 1997), Trans-Volga (Baskevich et al. 2008), and, finally, in the Far East (vicinity of Sovetskaya Gavan city in Khabarovsk Terr.; Kartavtseva et al. 2011). Since Malygin (1983), the findings of *Microtus rossiaemeridionalis* were assumed to follow generally the Transsiberian transport system, thus suggesting a human-induced way of introducing this vole eastwards. The reported occurrence of *Microtus rossiaemeridionalis* onOlkhon Island is one of the most eastern locations of the species and of particular interest, because it represents an isolated island population. Olkhon is the largest island of Lake Baikal (71 km in length and about 12 km in width or 730 km^2^) and has been geographically isolated for 0.7-0.8 million years (Galazij 2005, Agafonov and Akulov 2006). Considering the probable long autonomous existence of the vole population on the island, fixation of an unusual karyotype might have been expected, and this was in fact observed through a transformed X-chromosome (Fig. 2). Even if the voles were actually introduced within a historically short period, some dozens of years ago – coinciding with the age of the Transsiberian railway system – this observation adds to the little known intraspecies karyotypic variability in *Microtus rossiaemeridionalis*. Similar variation in the X-chromosome due to a reduced amount of heterochromatin has been reported for 54-chromosome voles from two geographically distant populations, i.e. one from western Siberia in the vicinity of Novosibirsk (Yakimenko and Kryukov 1997) and the other from the Arctic island of Svalbard (Fredga et al. 1990). In both those cases, a rearrangement was detected in a single X-chromosome of a sole specimen among a few studied individuals and interpreted as the deletion of a heterochromatic part in the X-chromosome (Fredga et al. 1990, Yakimenko and Kryukov 1997).

Meiotic preparations in a male from Olkhon Island (Fig. 3) revealed that the sex chromosomes remain asynaptyc which is typical for *Microtus rossiaemeridionalis* and the related species (Borodin et al. 1995, Mitsainas et al. 2010).

Cytogenetically, our findings indicate that in the *Microtus rossiaemeridionalis* karyotype, which otherwise is being considered rather stable, there is an X chromosome predisposition to intraspecific variation. Our data indicate that the variation affects the heterochromatic part of the X chromosome and have shown the value of karyotypic investigations on new and especially extreme localities in uncovering new karyotypic variability. Even in a rather invariant species like *Microtus rossiaemeridionalis*, such studies are worthwhile.
